# Detection of body shape changes in obesity monitoring using image processing techniques

**DOI:** 10.1038/s41598-024-73270-6

**Published:** 2024-10-15

**Authors:** Uçman Ergün, Elif Aktepe, Yavuz Bahadır Koca

**Affiliations:** 1 Engineering Faculty, Biomedical Engineering Department , Afyon Kocatepe University, 03200 Afyonkarahisar, Turkey; 2 Afyon Vocational School, Electronics and Automation Department , Afyon Kocatepe University, 03200 Afyonkarahisar, Turkey; 3 Engineering Faculty, Electrical Engineering Department, Afyon Kocatepe University, 03200 Afyonkarahisar, Turkey

**Keywords:** Obesity, Body measurement, Image processing, Biomedical, Mobile application, Biomedical engineering, Electrical and electronic engineering, Diseases, Health care, Computer science, Information technology

## Abstract

Body measurements are primarily made with a tape measure. In measurements taken with a tape measure, the inability to take measurements from the same part of the body each time, incorrect positioning of the tape measure, the occurrence of incorrect measurements, and the need for a person to take the measurements are significant problems in the traditional measurement method. Due to the social distancing rule that must be followed during the Covid-19 pandemic, the close contact between the person to be measured and the person taking the measurement became the starting point of this study. This study focuses on the detecting body shape changes using image processing techniques with 2D imaging. The novelty of the work is that non-contact body measurements are taken more accurately and reliably using the cosine theorem. Regular monitoring of obese patients is important in combating obesity, which is also the source of many health problems. In the monitoring of obese patients, it is necessary to determine the rate of slimming in areas where fat accumulation is intense. The error margin between the real measurements of human models and the calculated measurements was calculated as an average of ± 5.16% for waistline and an average of ± 4.58% for hip size. The cosine theorem was used instead of the ellipse formula used in the literature, and it was observed that the cosine theorem obtained results closer to reality. It is also thought that the developed system will be beneficial not only for extracting body measurements but also for extracting body measurements contactless in the textile sector. The study demonstrates the feasibility of image processing for non-contact body anthropometry and shape tracking.

## Introduction

Excess weight and obesity are defined as abnormal or excessive fat accumulation in the body, which poses a health risk^1^. Body mass index (BMI) calculation is used to determine whether an individual is underweight, normal weight, overweight, or obese. BMI is calculated by dividing the individual’s weight by the square of their height. According to the result, an individual is considered underweight if they have a BMI of less than 18.5, normal weight if they have a BMI between 18.5 and 24.9, overweight if they have a BMI between 25 and 29.9, and obese if they have a BMI of 30 or higher^2–4^.

In 2022, one in eight people worldwide were classified as obese. This translates to over 1 billion people. Of this number, 650 million are adults, 340 million are young people, and 39 million are children. Obesity cases are still increasing, and the World Health Organization (WHO) estimates that by 2025, approximately 167 million people will have their health negatively affected due to being overweight or obese. Obesity, as a disease, affects many systems in the body. It affects many organs, including the heart, liver, kidneys, joints, and reproductive system. It also causes the development of non-communicable diseases such as type 2 diabetes, cardiovascular diseases, hypertension, stroke, and various types of cancer. In 2019, it was estimated that obesity, extremely high BMI, and diseases affecting other organs caused approximately 5 million deaths. From this point of view, obesity also has serious economic effects. The global cost is estimated to be 3 trillion USD by 2030 and 18 trillion USD by 2060 ^5–7^.

The decrease in people’s mobility with the Covid-19 epidemic caused the number of obese patients to increase faster^8^. For obesity monitoring, it is important to follow the diet of the dietitian and analyze the patient’s weight loss. In this analysis, body measurements as well as the patient’s body weight should be used to determine the thinning in areas where fat accumulation is intense.

Anthropometry technique is used to take body measurements. Anthropometry is a scientific field that deals with the measurement of the human body. General anthropometry is an approach that includes all processes of data collection, documentation, summarization and analysis^9^. This method is used to numerically describe factors such as lengths, widths, heights, sizes and general body shape^10^. Anthropometry is used in various fields such as medicine, fitness, and the fashion industry^11,12^. Anthropometric measurements play an important role in monitoring the growth and development of individuals, determining the proportions between body parts, and monitoring body composition and adequate nutrition^13,14^.

Tape measures are commonly used for anthropometric measurements but have some limitations. These limitations include inconsistency in placement, the potential for human error, the need for close physical proximity between the measurer and the subject and growing social unease with close contact due to recent epidemics like that Covid19. Therefore, alternative measurement methods are being explored to address these shortcomings.

3D (three-dimensional) scanner systems can be used to automatically obtain human body measurements as an alternative to the traditional measurement method using a tape measure. Body measurements are standardized by defining measurement postures and body landmarks, ensuring comparability and repeatability. Studies show that the traditional measurement method and 3D scanners have similar accuracy levels and that 3D scanners are generally better in terms of repeatability^15–18^. Another advantage of using 3D scanners is the high speed of measurement. Automatic scanning can usually be completed in a few seconds or less. For high-quality scans, this time can go up to 30 s. The first commercial 3D body scanners emerged in the 1990s, but they were expensive and required trained personnel and extensive processing^19^. Today, scanning technology can offer similar performance compared to traditional measurement methods^20–22^. However, their accessibility may be limited due to their high cost.

Body measurements can be obtained using 2D (two-dimensional) photographs, which is an alternative to 3D scanners. In this technique, photographs of the individual are taken from the front and side. The images captured are used to extract the silhouette of the human body using image processing techniques such as edge detection algorithms. The sizes of the desired regions, such as the chest, waist, and hips, are calculated on the obtained silhouettes using the ellipse circumference formula. This method has been investigated and used in several studies^23–28^.

In this study, anthropometric measurement methods using 2D images were preferred as they are easy and do not require any cost. This approach offers a simpler alternative to traditional tape measure usage or 3D scanners that require advanced technology. It aims to obtain all body measurements by the individual taking their own photo with a mobile phone, without the need for another person. The goal is to obtain the individual’s body measurements using image processing algorithms. Background cleaning and edge detection algorithms were used to process the captured images.

The literature generally uses photographs taken from the front and side with a 90-degree difference. However, since the body shape does not resemble a calculable geometric shape such as an ellipse or a circle, studies performed with only front and side photographs may cause calculation errors. In this study, more photographs were used to achieve more realistic measurements. The body measurements of the individual can be calculated with a certain error rate thanks to these images processed with image processing techniques. This method shows that the results of studies conducted on human models can also be valid for measurements to be made on humans.

The study suggests that the obtained body measurements can provide opportunities for many useful applications not only in the healthcare field but also in other industries such as textiles. With the increasing shift towards e-commerce during the pandemic, some individuals experience difficulties in finding clothes that fit properly, especially in the textile sector, due to the different body measurements of each individual. Determining the body measurements of the individual with this method and enabling companies to make custom designs will put companies one step ahead in a competitive market in the textile sector.

## Related work

In today’s world, anthropometric measurements of the human body are generally taken using tape measure. A tape measure is a length measuring tool typically 15 mm wide and graduated in mm^29^. It is used to take height and sizes measurements of the body. When taking measurements with a tape measure, tight-fitting clothing that fits the body should be preferred. The tape measure should be held firmly during measurement but should not be squeezed so much as to distort the measurement. It is recommended to use centimeter tape measures at 1 mm intervals for circumference measurements^30^ The result is rounded to the nearest upper value in 1 mm^29^. However, tape measure measurements can be inaccurate due to factors such as the inability to take each measurement from the exact location and the incorrect positioning of the body. Additionally, there are problems such as the need for a person to take the measurement, the close contact between the patient and the person taking the measurement, and the potential for the measurement results to be inaccurate. Ultrasonic distance sensors, 3D scanners and 2D photo measurement methods are used to address these issues^31,32^.

3D scanners are used in the fashion and garment industry for various tasks such as body measurement, numerical data detection, visual data results, body analysis, product development, and proper clothing selection. 3D scanners are used to create a 3D model of an object^33^. The object is either placed on a rotating mechanism and scanned by rotating it 360 degrees, or a fixed 3D scanner rotates around the object, and a 3D model is obtained. The resulting 3D model can be printed out in the same size, smaller, or larger size using a 3D printer. Different studies have been conducted, such as reviewing mobile body scanning applications for clothing, evaluating the accuracy of anthropometric data from entry-level 3D handheld scanner and creating garment patterns with acceptable errors, 3D scanning technology for Additive Manufacturing (AM) digital models^34–36^.

The first market systems were scanners with limitations such as closing cavities (e.g., belly). Later, most systems became laser-based. In these systems, a line is projected from top to bottom in about 20 s and scanned. A camera captures the image at a certain angle and calculates the object’s distance to the camera using trigonometry. Typical laser scanning systems include Vitronic, Cyberware and Hamamatsu^37^.

In a study by Ly et al.^38^, a device was designed to estimate human body dimensions using an ultrasonic sensor SRF-04, a low-cost and efficient device. A device was designed using ultrasonic sensors. Based on ultrasonic sensor arrays, the intelligent body measurement system efficiently captures chest, waist and hip measurements and is ideal for automatically determining trouser size. In the study, 99.61% measurement accuracy was achieved for chest, waist and hip information^39^. Anthropometric measurements were estimated by deriving ellipse equations based on 2D-based body shape variations^40^.

As stated by Ashdown et al.^41^, scanners for the fashion industry can capture the correct 3D relationship of a garment to the body by minimizing visual distraction. The individual’s 3D model is obtained by placing the individual or the 3D scanner on movable mechanisms that rotate 360 degrees. The individual’s body measurements are extracted from the obtained model. The individual can see the suitable garment on themself through virtual fitting rooms. The 3D image allows for a quick assessment of the validity of the scan. Dimensions for different clothes can be used without re-measuring. The measurements obtained using this technology can be more accurate and repeatable than those obtained with traditional and physical measurements. Measurement data can be refreshed or revised as needed^42^. However, some 3D scanners are not preferred due to their large size and high cost.

Seo et al.^43^ prepared a dataset of 19 volunteers to investigate the possibility of automatically measuring waistline using a Kinect sensor. The depth images of each individual were recorded and the actual measurements were recorded by a medical professional using tape. The study presents a new method of waist measurement using SVM regression. Spahiu et al.^44^ conducted a study in which they extracted anthropometric data from 3D body models using a 3D laser scanning system called Konica Minolta VIVID 910. The results showed that the method was effective in accurately extracting body measurements.

Mobile 3D body scanning technologies have also advanced to help customers choose the appropriate clothing size recently. Researchers investigate 3D mobile body scanning applications by determining their methods, features and advantages^45^. The mobile application called Nettelo was examined to extract body measurements using 3D scanning. This free mobile application has the features of being able to perform 3D body scans using the camera of the mobile device and analyze the scanned body. However, the tests showed that the application did not perform as desired and was not successful^36,46,47^.

Lin and Wang^28^ proposed a method for automatically extracting body features using 2D images. This method yielded 60 feature points. It was stated that the extracted feature points could be used further in various applications, such as automating body size measurements. The study by Ouellet and Michaud^48^ expanded on the work of Lin and Wang (2011) by developing an algorithm to extract body features from a two-dimensional image automatically. This algorithm uses 45 front and 24 side feature points from a 2D image in front of a black background to extract 20 front and 13 side measurements. This study offers a more effective solution by improving the number of feature points and processing time.

Luo’s^24^ study aimed to summarize 2D image-based contactless anthropometry and solve the problem of measuring body size dimensions in clothing e-commerce. The study describes a three-step process for obtaining 2D image-based contactless anthropometric values: image silhouette extraction, characteristic body measurement, and perimeter calculation. The study concluded that measurements obtained from photographs have high accuracy and reliability compared to traditional measurement methods.

While 3D body scanning systems can provide accurate results, their use in industry, especially in small business settings, is limited. The study extracted body dimensions by taking 13 measurements. These measurements were calculated from neck, shoulder, chest, chest underbust, waist, hip, lower hip, thigh sizes, torso length, body height, knee height and waist height. The experiments were conducted on the front and side 2D images. As a result of the experiments, sizes showed a higher correlation than height measurements. The study recommended further research on body height measurements^25^.

Waist circumference measurements may have a margin of error of ± 2–3 cm due to anatomical structure changes. This leads to a 3–5% error rate, affecting the accuracy of assessing abdominal obesity^49,50^. CT and manual measurements have a strong correlation between waist circumference. This provides a method for identifying abdominal obesity with a potential 3–5% error rate improvement^51^.

Tape measures are a simple and affordable tool for taking body measurements. However, they can be inaccurate due to user error and difficulty reaching all body locations. 3D body scanners offer a more accurate and objective way to measure the body, but they are expensive and not widely available. 2D photogrammetry is a promising new technology that can take precise body measurements from photographs. This technology is still under development, but it has the potential to be a more affordable and accessible alternative to 3D body scanners. The best body measurement method depends on your needs and resources. A tape measure may be sufficient if you need a quick and easy way to take basic measurements. A 3D body scanner or 2D photogrammetry system may be a better option if you need more accurate measurements for clothing or medical purposes.

## Methodology

In this study, 16 prototype human models were produced using a 3D printer and measurements were taken of the models. Prototype human models were printed on a 3D printer on a 1/16 scale, like actual human size. In addition, different BMI characteristics were defined and produced for prototype models. A mobile application-controlled turntable was designed to rotate the models at set angles. Through this mobile application, the turntable can be controlled and the images taken of the models can be uploaded to a database. The uploaded photos are then analyzed using image processing techniques in a simulation program. Waist and hip sizes of the models were calculated using cosine theory and the ellipse formula. The study consists of developing the mobile application, designing and manufacturing the turntable, image processing and comparative analysis.

### Mobile application

There are many alternative methods for developing mobile applications. Block-based and non-block-based mobile application development platforms are available. Block-based platforms make writing code easier and save time by providing a puzzle structure structured with blocks of code. In this study, the Kodular platform was preferred for data infrastructure.

**Fig. 1:**
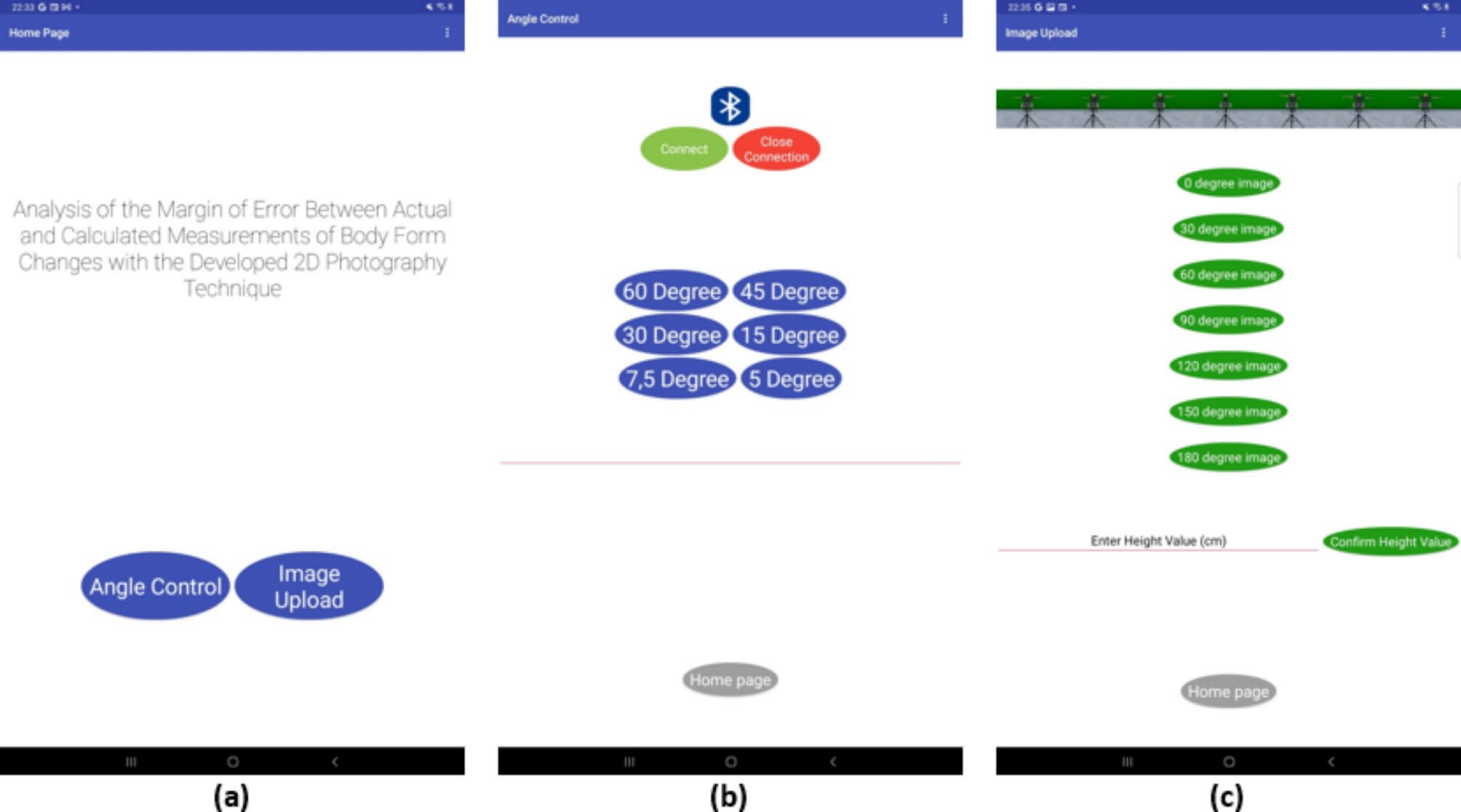
Developed mobile application. (**a)** Main screen. (**b)** Control. (**c)** Data transfer and saving.

**Fig. 2:**
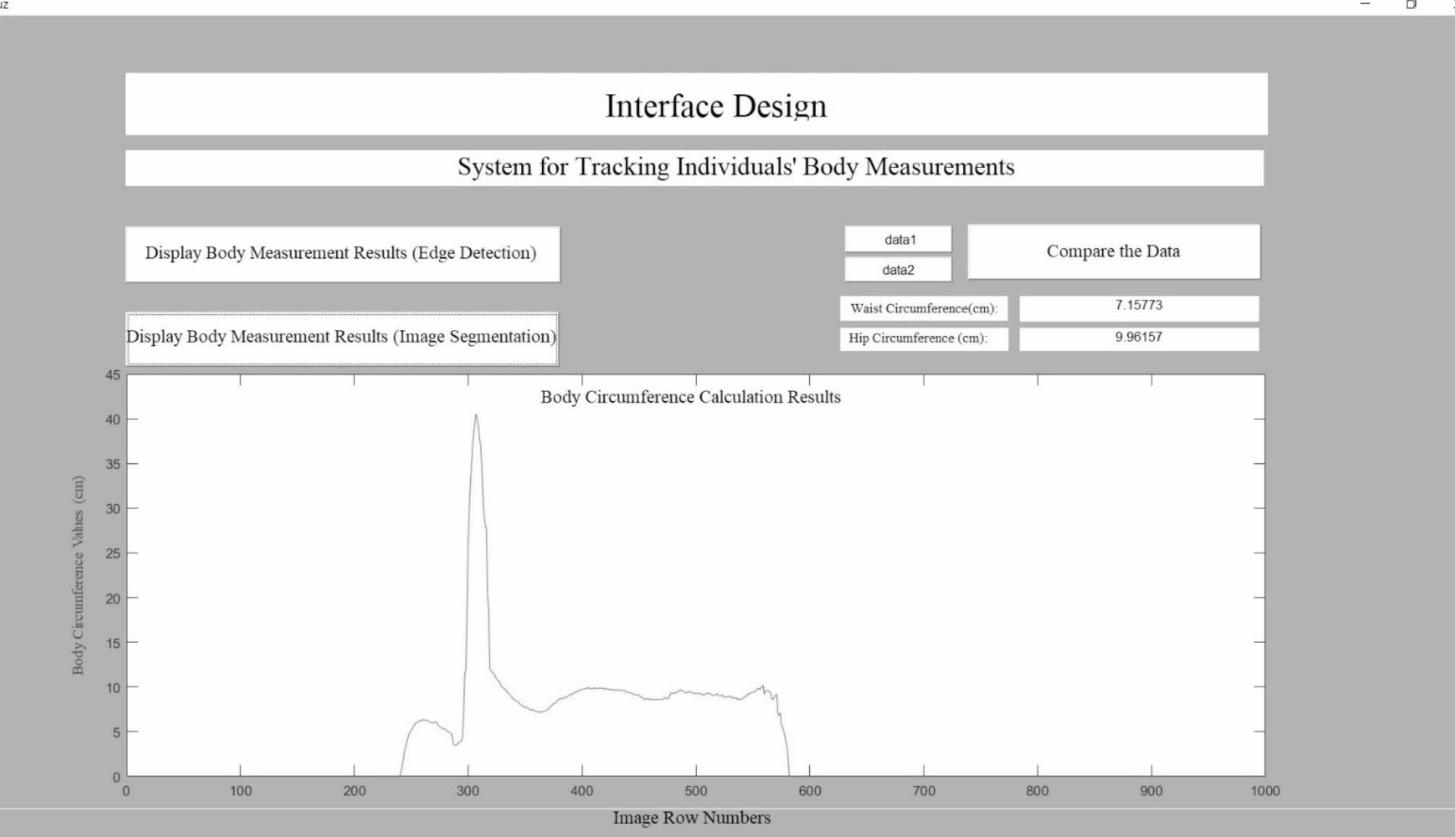
Interface developed in the simulation program.

One of the purposes of the developed mobile application is to upload the captured images to the database. Another purpose is to control the movement of the turntable designed for prototype human models at certain angles through the mobile application. There are three windows in the mobile application. The first window is the main screen page in Fig. 1a. There are “Angle Control” and “Image Upload” options on this screen. Figure 1(b) shows the Angle Control screen opened as the second screen. This screen contains a ListPicker, a TextBox and eight buttons. The ListPicker lists available Bluetooth connections, while the TextBox displays the angle at which the stepper motor is rotated. The buttons have functions for toggling Bluetooth connection, controlling angles at specific degrees, and returning to the home screen. In Fig. 1c, the third screen, the Image Upload screen, is shown. There are nine buttons and one TextBox in this section. While the buttons enable the selection of images at different degrees, TextBox is used to enter the actual height value of the human model in centimeters. The received images are uploaded to the Cloudinary database. At the same time, the URL addresses and height values are saved in the Firebase database. Firebase database is a real-time database used by the mobile application (Figs. 1 and 2).

This way, the mobile application obtains users’ upload rates and saves them in databases such as Firebase and Cloudinary. The Home Page button takes the user back to the start screen.

### Mobile app controlled rotating table design

The turntable is designed to rotate 3D printed human models at certain angles. The design was carried out in the 3D solid modeling program. Angle control of the turntable is provided through the developed mobile application. Communication between the mobile application and the turntable is carried out using a microcontroller-based Bluetooth module.

The turntable prototype was designed with a diameter of 16 cm and a height of 3 cm. The energy of the system in the table was provided from the USB port. A part that can be attached to the moving end of the stepper motor to fix 28 BYJ-48 Geared Stepper Motor on the turntable has also been designed in 3D. The printouts of the designed models were taken using tinylab 3B 1.75 mm white PLA (Polylactic Acid) filament using a Creality 3B Ender 3 Pro 3D printer. The table control system, developed on a microcontroller basis, works synchronously with the mobile application, allowing the table to rotate at the desired rotation angles and saving the captured images to the database.

### Image processing

**Fig. 3:**
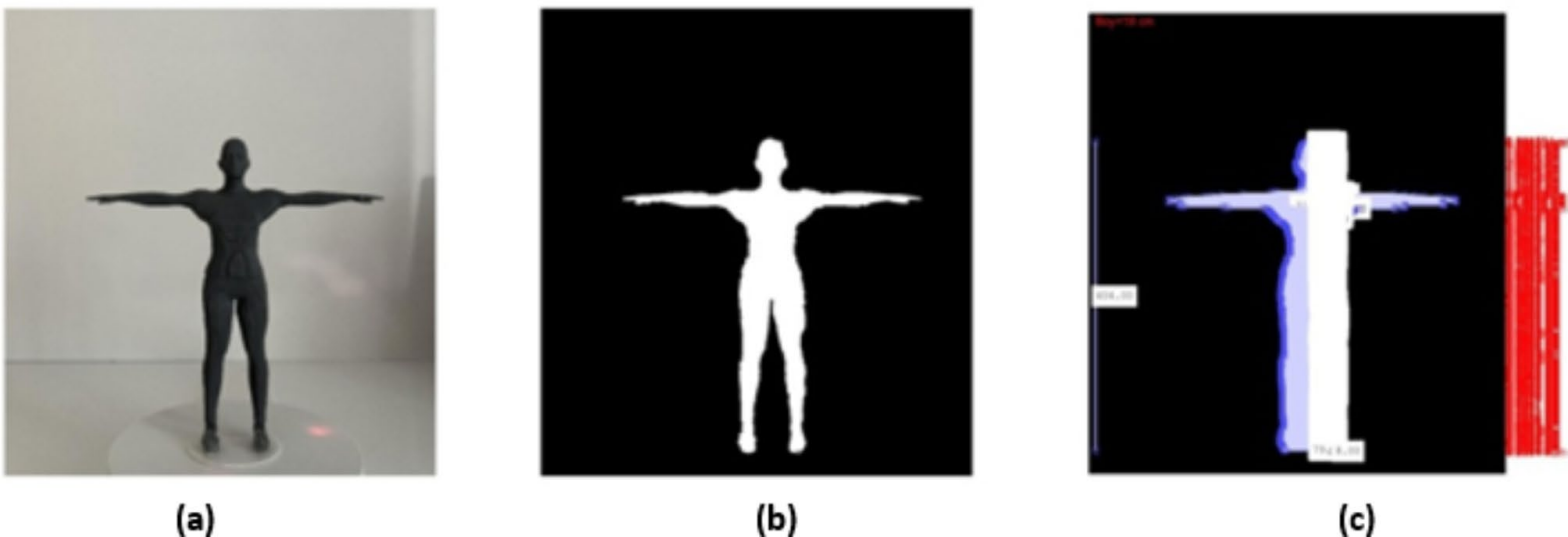
Image processing steps. (**a**) Initial image. (**b**) Background cleared image. (**c**) Calculated width values image.

While acquiring images of prototype human models, a white background was used, upright positioning of the developed prototype models and placement at the centre of the turntable. During the image capture process, attention was paid to positioning the mobile device on a tripod in an upright position, as shown in Fig. 3 and maintaining a distance of 30 cm between the mobile device and the prototype model. In Fig. 3a shows the first image taken during the image processing. Figure 3b shows the cleared background of the captured image and Fig. 3c shows the image of the calculated value.

Under the specified conditions, images of prototype models placed at the centre of the turntable were captured at 30-degree intervals. The captured images are then uploaded to the cloud database. The URL extensions and dimensional values of the uploaded images are also saved to the Firebase database. The function obtained using the Matlab Image Segmentation application begins with the image being loaded into the application. A mask is created by selecting the foreground and background of the image. Then, the application prepares the image with the background cleared for further processing. This background-cleared image or function can be exported. The prepared function is integrated into the first part of the measurement extraction algorithms developed in the simulation program. The colour image is converted to grayscale. The Canny edge detection algorithm is applied to the grayscale image to identify the human silhouette. This algorithm is preferred as it is widely used in the literature^25,28,48,52^. Using the developed measurement extraction algorithms, all body measurements are obtained. These measurements are recorded using the cosine theorem and the ellipse formula for the perimeter calculation of each row. The interface developed in the simulation program compares the measured anthropometric old and new values. This interface allows users to analyze and compare measurement data visually. Measurement data can be managed more easily and quickly through the interface provided in Fig. 2.

Calculations regarding waist width and length for the developed prototype models are given in Eqs. (1) and (2), respectively.

1 $${\text{AWW}}\left( {{\text{cm}}} \right)=\frac{{{\text{AHV}}\left( {{\text{cm}}} \right){\text{*CWW}}\left( {{\text{px}}} \right)}}{{{\text{CHV}}\left( {{\text{px}}} \right)}}$$

2 $${\text{AWL}}\left( {{\text{cm}}} \right)=\frac{{{\text{AHV}}\left( {{\text{cm}}} \right){\text{*CWL}}\left( {{\text{px}}} \right)}}{{CHV\left( {{\text{px}}} \right)}}$$

Here, AWW = actual waist width (cm), AWL = actual waist length (cm), AHV = actual height value (cm), CWW = Calculated Waist Width(px), CWL = Calculated Waist Length(px), CHV = Calculated Height Value (px)..

**Fig. 4:**
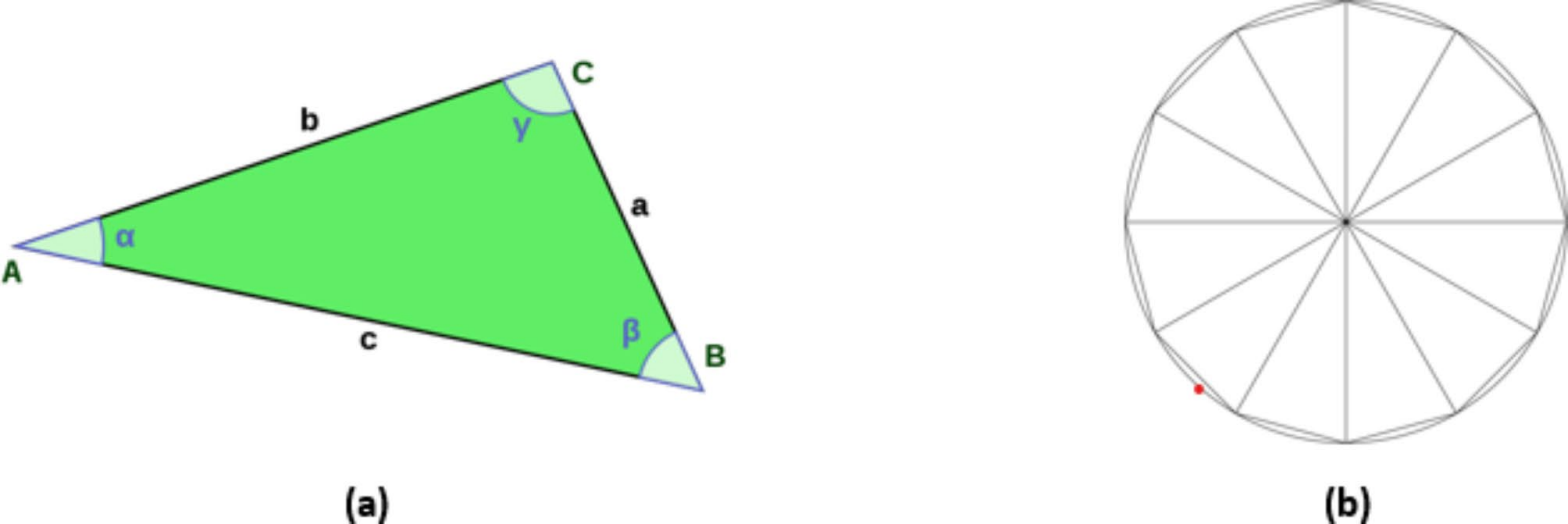
(**a**) Application example of cosine theorem in triangle. (**b**) 12 triangles of 30 degrees placed in a circle.

In the studies conducted, analyzes were generally carried out using ellipse circumference calculations using actual waist length and width values^25^. The ellipse calculation given in Eq. (3) was used in the studies. However, in this study, the cosine theorem was used instead of the ellipse perimeter formula in order to minimize the error. Necessary calculations were made using the triangle application example given in Fig. 4a.

3 $${\text{Ellipse}}\;{\text{Circumference}}=2{{{\uppi}}}\sqrt {\frac{{{{\text{a}}^2}+{{\text{b}}^2}}}{2}}$$

4 $$\begin{array}{*{20}c} {{\text{a}}^{2} = {\text{b}}^{2} + {\text{c}}^{2} - 2{\text{*b*c*cos}}\left( \alpha \right)} \\ {{\text{b}}^{2} = {\text{a}}^{2} + {\text{c}}^{2} - 2{\text{*a*c*cos}}\left( \beta \right)} \\ {{\text{c}}^{2} = {\text{a}}^{2} + {\text{b}}^{2} - 2{\text{*a*b*cos}}\left( {\text{y}} \right)} \\ \end{array}$$

**Fig. 5:**
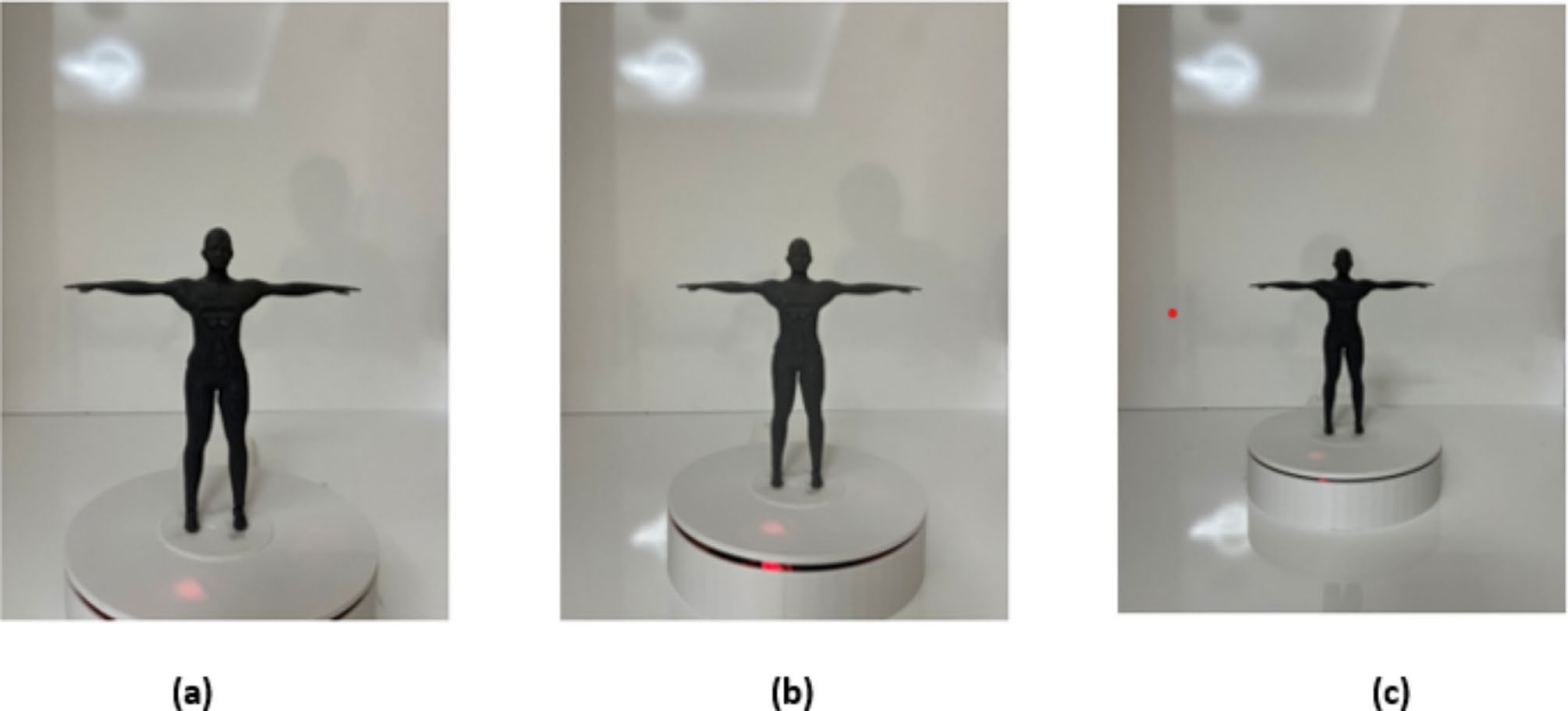
Images of the human model number M12 taken from different distances. (**a**) 25 cm. (**b**) 30 cm. (**c**) 40 cm.

Calculations regarding the cosine theorem are given in Eq. (4). When the calculation is made using the cosine theorem, a dodecagonal shape is obtained at 30 degrees. When using the cosine theorem, two sides and the angle value between them must be known in order to find the measure of the third side. Therefore, it is known that the prototype models in the study were rotated at 30-degree angles. Thus, the third side can be calculated. If the same process is performed for 7 degrees, 6 edges are obtained. The half perimeter can be calculated by adding these edge values and the full perimeter is found by taking twice the half perimeter value Figure 4b. In the picture, 12 triangles of 30 degrees can be seen placed inside the circle. Thanks to this method, errors caused by the ellipse formula in body size are minimized. The margin of error in calculations based on this is found to be 1.02%. One of the reasons why the cosine theorem is preferred is that its margin of error is quite low. Thanks to this method, the results obtained are more accurate and reliable (Fig. 5).

## Results

The study trials were carried out on 1/16 scale prototype human models. Sixteen prototype human models were printed using a 3D printer. Five of these models were printed in the same size, allowing comparison. The five printed models were placed in the center of the turntable, and photographs were taken from different distances and angles with the wide camera system of the iPhone 13 mobile device. The pictures were processed and analyzed, and the error rate was calculated by comparing the computed results with the actual values. The average error rate was calculated by taking the square roots of the sum of the squares of the error rates obtained.

Evaluations of 16 developed prototype models were made. The actual measurements of the models were obtained from the 3D printer program. In addition, the actual measurements of the models were checked manually with a tape measure. The distance between the turntable and the mobile device was set to 30 cm and images of the models were taken at 30-degree intervals. The images were processed and the results were recorded. The error rate between the calculated results and the actual values was calculated as a percentage, and the average of the square roots of the sum of the squares of the error rates and the average error rates were calculated as a percentage. As a result of the calculations, the average error rate for waistline given in Table 1 was calculated as 5.16% and for hip size as 4.58%.

**Table 1:** Comparison of waistline and hip size of prototype models.

Model	Height (cm)	Waistline actual value (cm)	Waistline measured value (cm)	Error (%)	Hip size actual value (cm)	Hip size measure value (cm)	Error (%)
M 1	18	8.5	8.16	4.02	11.5	11.38	1.02
M 2	9.8	4.5	4.20	6.69	6	5.94	0.97
M 3	10	6	5.91	1.55	8	7.70	3.77
M 4	10	4.7	4.60	2.15	6.5	6.09	6.35
M 5	11	7.5	6.97	7.09	8	7.64	4.53
M 6	18	8	7.81	2.43	11.5	11.48	0.20
M 7	16.5	8	7.57	5.40	11	10.93	0.63
M 8	14	5.5	4.97	9.56	8.5	8.07	5.11
M 9	14	4	3.91	2.37	6.5	6.68	2.74
M 10	11	5	4.85	2.91	7.3	6.74	7.66
M 11	11	4	3.67	8.16	5	4.97	0.61
M 12	15	6.1	6.08	0.26	8.1	8.76	8.20
M 13	15	7.1	6.60	7.04	9.2	9.07	1.45
M 14	15	6.6	6.58	0.28	8.6	9.32	9.60
M 15	15	7.3	6.90	5.46	9.8	10.01	2.10
M 16	15	7.6	7.35	4.58	10.1	10.08	0.17

### Evaluation of images based on distance

The developed prototype models were printed with a 3D printer and placed on the turntable. Images of the placed models were taken from distances of 25 cm, 30 cm and 40 cm, respectively, as seen in Fig. 5. Seven images were taken for each model.

According to the comparison results, the average percentage error rates for waistline were measured as 4.59% for 25 cm, 2.43% for 30 cm and 3.45% for 40 cm. Average percentage error rates for hip size were calculated as 6.30% for 25 cm, 5.27% for 30 cm and 5.57% for 40 cm. Waistline values obtained from images taken from different distances are presented comparatively in Table 2, and hip size values are presented comparatively in Table 3. It can be seen that the pictures with the lowest error rates in both waistline and hip size calculations were taken from a distance of 30 cm.

**Table 2:** Waistline values taken from images taken from different distances.

Model No	Waistline (cm)	25 cm	Error (%)	30 cm	Error (%)	40 cm	Error (%)
M12	6.1	6.00	1.62	5.00	1.70	6.33	3.84
M13	7.1	6.65	6.37	6.81	4.09	6.66	6.18
M14	6.6	6.55	0.69	6.66	0.85	6.59	0.22
M15	7.3	6.83	6.50	6.94	4.97	7.01	4.03
M16	7.6	7.27	4.39	7.30	4.02	7.43	2.29

According to the comparison results, the average percentage error rates for hip sizes are as follows.

**Table 3:** Hip size values taken from images taken from different distances.

Model No	Hip size (cm)	25 cm	Error (%)	30 cm	Error (%)	40 cm	Error (%)
M12	8.1	8.81	8.80	8.98	10.90	8.85	9.25
M13	9.2	9.29	0.96	9.47	2.95	9.37	1.84
M14	8.6	9.52	10.67	9.49	10.38	9.29	8.03
M15	9.8	9.56	2.47	9.73	0.70	9.67	1.32
M16	10.1	10.06	0.43	10.11	0.09	10.14	0.37

**Fig. 6:**
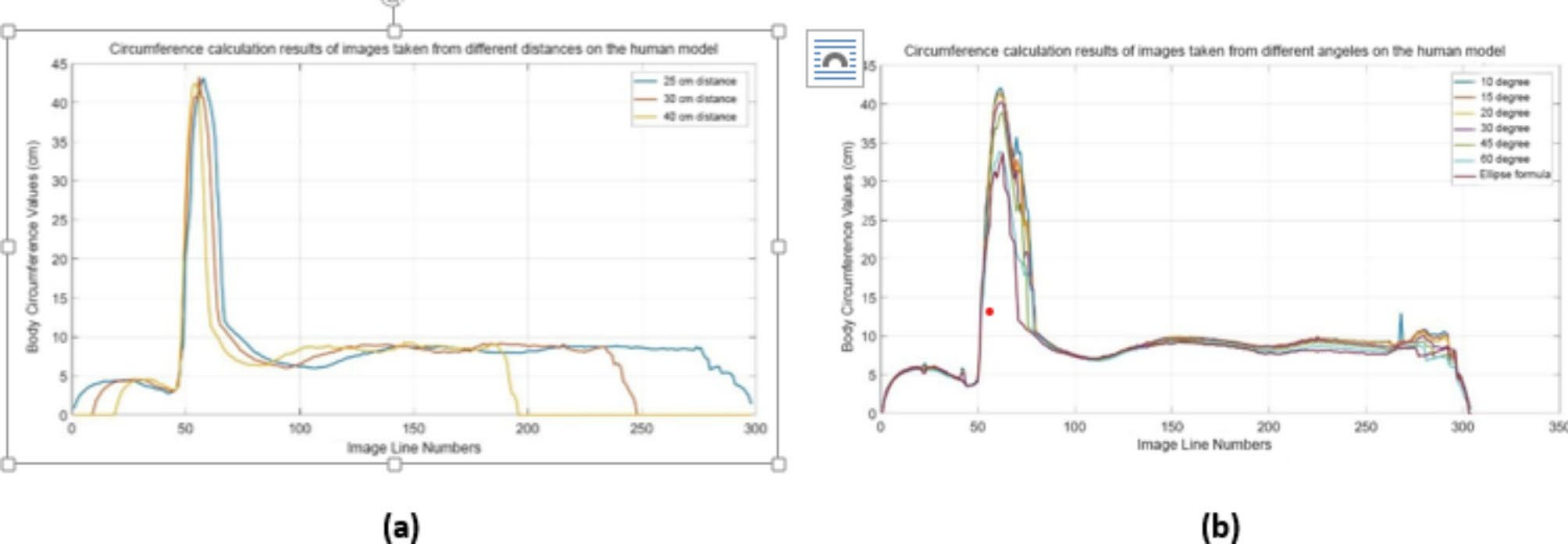
(**a**) Circumference calculation of images taken from different distances on the model. (**b**) Circumference calculation results of images taken from different angles on the model.

For this reason, the distance in all images taken from the prototype models developed within the scope of the study was taken as 30 cm. The environmental values obtained in this way are closer to reality. After these images are processed, the calculations of the waist circumference are given in Fig. 6a.

### Evaluation of images taken at different degrees

Within the scope of the study, an evaluation was made for images taken from different degrees. For the human model numbered M15, which is a prototype model developed in this context, images were taken at angles of 10, 20, 30, 45 and 60 degrees, respectively. In this context, 19 photographs were recorded for 10 degrees, 10 photographs for 20 degrees, 7 photographs for 30 degrees, 5 photographs for 45 degrees and 4 images for 60 degrees. The images taken were processed with the software developed in the Matlab simulation program and waistline and hip size calculations were made for each angle using the cosine theorem. Additionally, these images were compared with ellipse calculation. The results of waistline and hip size calculations for images taken from different angles for a prototype model are given in Fig. 6b.

**Fig. 7:**
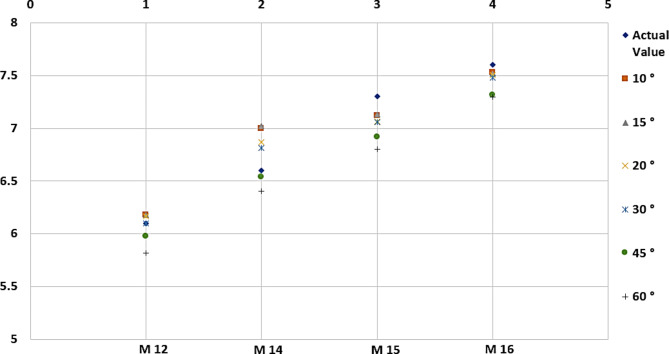
Various degrees of error deviation based on actual waistline.

**Fig. 8:**
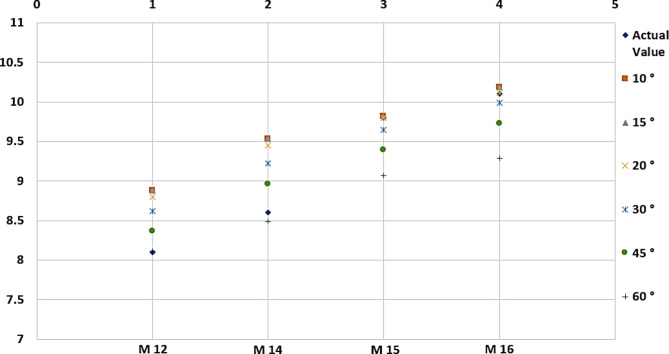
Various degrees of error deviation based on actual waistline.

Tables 4 and 5 show that the average error values in the waistline and hip size measurements differ. It was observed that the average error value for the waistline was lower at 30 degrees, but the average error value for hip size was lower at 45 degrees. According to these results, measuring the waistline at 30 degrees in human trials was preferred because fewer errors in waistline measurement will provide an advantage in accuracy and reliability. In addition, graphical representations showing the margins of error corresponding to the degrees specified in Tables 4 and 5 are shown in Figs. 7 and 8, respectively.

**Table 4:** Comparison of waistline values taken at different degrees.

Model No	Waistline (cm)	10°	15°	20°	30°	45°	60°
M12	6.1	6.18	6.18	6.16	6.10	5.98	5.89
M14	6.6	6.99	7.02	6.87	6.81	6.54	6.41
M15	7.3	7.12	7.13	7.07	7.06	6.92	6.80
M16	7.6	7.53	7.52	7.51	7.48	7.31	7.30

**Table 5:** Comparison of hip size values taken at different degrees.

Model No	Hip size (cm)	10°	15°	20°	30°	45°	60°
M12	8.1	8.88	8.88	8.80	8.69	8.37	8.12
M14	8.6	9.53	9.53	9.45	9.22	8.96	8.49
M15	9.8	9.82	9.88	9.80	9.64	9.39	9.06
M16	10.1	10.19	10.19	10.12	9.99	9.73	9.28

#### Evaluation of images thin and overweight models

Waistline of models printed as thin and overweight were compared with actual measurements. While the exact difference in waistline was 1.3 cm between the thin and overweight models, the calculated difference was 1.48 cm. According to the interpretation of Table 6, the actual and computed differences in the waist area of the model were close to each other. This result shows that waistline measurements of models printed with a 3D printer are pretty accurate and reliable. The fact that the calculated difference between the waistline of thin and overweight models is very close to the real difference shows that the models obtained by 3D printing accurately represent the waistline.

**Table 6:** Comparison of waist circumferences for underweight and overweight individual models.

Models	Waistline actual (cm)	Waistline measured (cm)
Skinny	4.7	4.47
Overweight	6	5.95
Differ	1.3	1.48

**Fig. 9:**
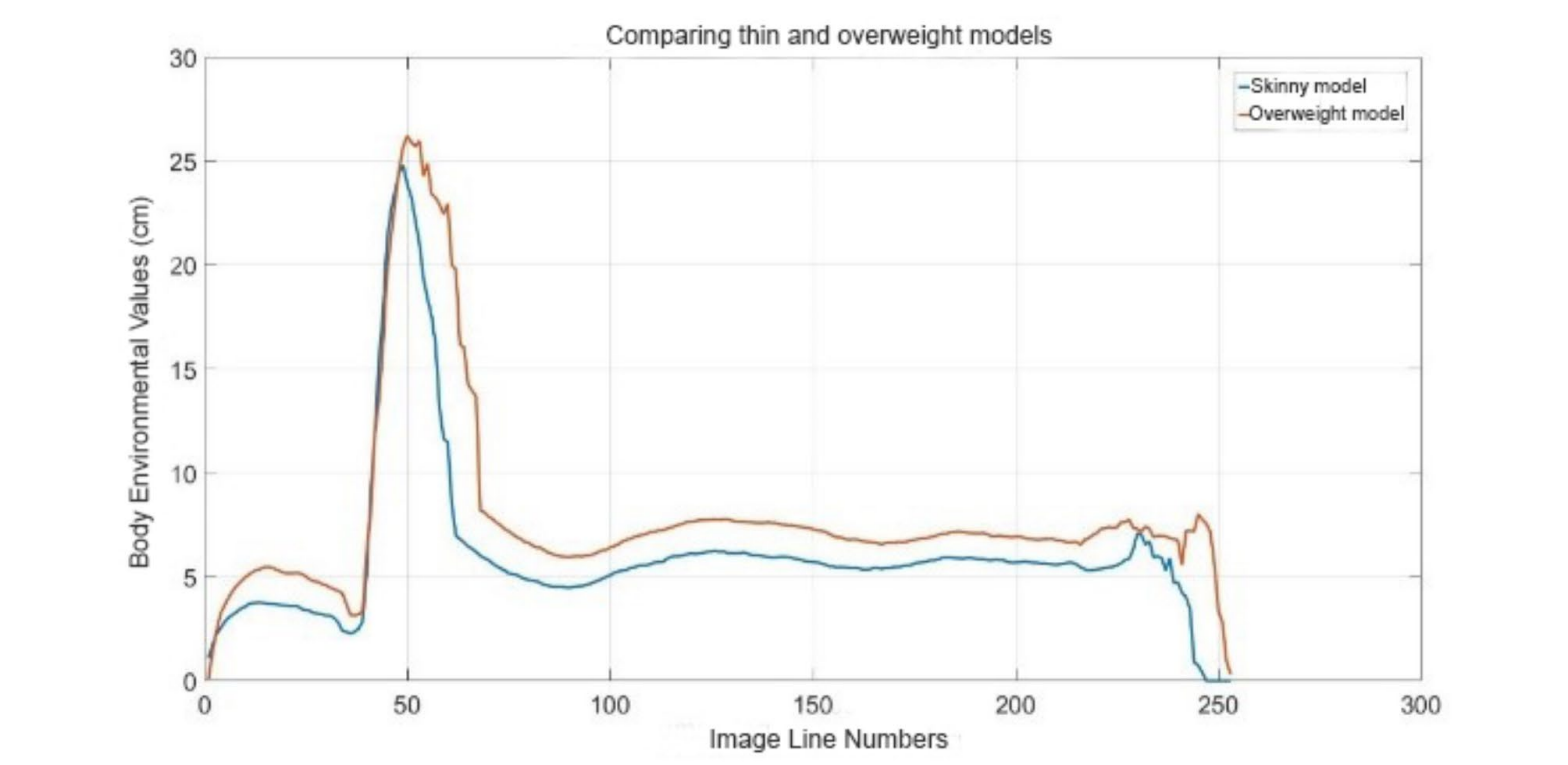
Comparison thin and overweight models.

Figure 9 shows the comparison graph of thin and overweight models’ whole body size measurements. This chart visually indicates how the body measurements of both models differ. In summary, the study observed that the waistline measurements of thin and overweight models printed with a 3D printer were compatible with actual measurements and gave accurate results.

According to the calculations, the image’s width of the 15 cm tall model taken at 0 degrees was calculated as 0.75. When the same process was repeated at 30 degrees, the width value was found to be 0.71. When the cosine theorem is applied to calculate the difference between these two values, the value found is 0.38. This process was repeated in the same way at intervals of 30 and 60 degrees, 60 and 90 degrees, 90 and 120 degrees, 120 and 150 degrees, and 150 and 180 degrees, and the values obtained for each interval were added and multiplied. As a result of this calculation, the total environmental value within 180 degrees was found to be 4.14. The resulting calculations are given in Table 7. For the same model, the circumference was calculated using the ellipse formula. The ellipse formula uses waist width values at 0 and 90-degree angles as radii. For 0 degrees, half of the waist width is calculated as 0.75, and for 90 degrees, half of the waist width is calculated as 0.56. These two values are defined as the long and short radii of the ellipse. When the circumference of the ellipse was calculated, the value was found to be 4.13.

**Table 7:** Application results of images taken at 30-degree angles.

Degrees	0–30 (cm)	30–60 (cm)	60–90 (cm)	90–120 (cm)	120–150 (cm)	150–180 (cm)
Cosine	0.39	0.36	0.31	0.30	0.35	0.38

## Discussion

The image processing algorithms used in the study were designed to adapt to the structure of the human body of different sizes and shapes. Initially, surveying human models is a practical starting point for verifying the basic principles and algorithms of the system. The developed algorithms have shown high accuracy rates on various models. This accuracy rate is also anticipated to be maintained for real people.

McCarthy et al.^53^ conducted a statistical review comparing anthropometric measurements made with mobile phones with professional systems. This study, which they conducted on real people, revealed the success of measuring mobile phones. This shows that the method and model used in our research is an accurate approach.

When conducting studies on people, the parameters that will increase the error rate may be the loose clothing that people can wear and posture disorders. This situation may increase the error rate compared to models. In the study conducted by Foysal et al.^54^, it is seen that the measurements of real people photographed were made with clothes. This shows us that there may be deviations from the actual values. From this perspective, it is evaluated that the measurement made on the model will show a more accurate approach. Studies^49–51^ stated that the margin of error was between 3 and 5%. In our research, it is seen that these rates are within the limits. In addition, Jiang et al.^55^ stated that internal tissue analysis with physical parameters such as gender, age and race may vary among different populations. This approach gives motivation future studies aim to work on an approach integrating biological sensors with more advanced technologies.

The studies of^9,21,24,26,41,54^ are limited to body extraction only in the textile sector. This study also allows regular monitoring in the fight against obesity in the textile and health sectors.

The ellipse theorem was preferred in the studies like^23–28,54^. Another important innovation is that specific body measurements were obtained with higher accuracy using the cosine theorem in our research. Ellipse and cosine theorem comparisons were performed on the prototype numbered M16.

As a result of the comparison, it was seen that the values obtained for waist circumference were close to the results calculated by both methods. However, it has been observed that there are more significant differences between the results calculated by the ellipse formula and the cosine theorem for hip circumference. Table 8 shows the values of perimeter calculations made using the cosine theorem and ellipse formula.

**Table 8:** Comparison of the results of applying the cosine theorem and the ellipse formula.

	Waistline (cm)	Hip size (cm)
Actual value	7.60	10.10
Cosines theorem	7.29	10.11
Ellipse formula	7.22	9.34
Cos error ratio (%)	4.02	0.09
Ellipse error ratio (%)	5.05	7.51

**Fig. 10:**
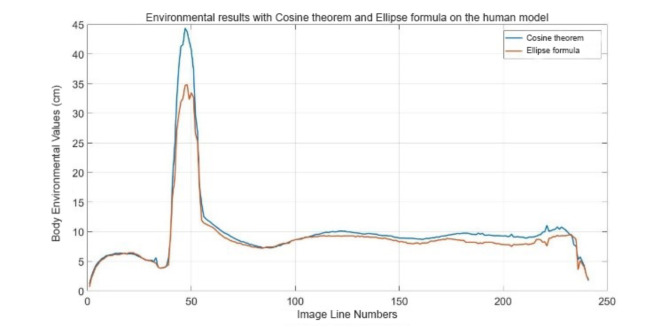
Graph of perimeter results with cosine theorem and ellipse formula.

As can be seen in Table 8, both methods give different results for waist and hip circumferences. As a result, it was observed that the cosine theorem used in the study obtained results closer to reality than the ellipse formula used in the literature. As stated in the previous sections, it can be seen that results closer to reality can be obtained by taking seven images instead of 2 images using the cosine theorem. The comparison results graph of the cosine theorem and ellipse formulas for the prototype model numbered M16 is shown in Fig. 10.

## Conclusion

This study considered the increase in COVID-19 due to different factors in recent years and the importance of monitoring obesity. A new technique has been developed to overcome the difficulties in taking traditional body measurements. In the study, individuals’ body measurements were compared with measurements taken using 2D photographs and tape measure. The 2D photography technique involves taking seven photographs and extracting measurements using the cosine theorem. The average error rates of the error rate values calculated for the waist and hip circumferences of the developed prototype models were determined as percentages. The average error rate was calculated as 5.16% for waist circumference and 4.58% for hip circumference.

The ellipse formula used in the literature was compared with the cosine theorem used in the study, and it was observed that the cosine theorem used in this study obtained results closer to reality.

The developed system was advantageous as it made body measurements contactless and with low error rates. However, when working with real people, the fact that seven photographs are required to take 2D photographs and that some individuals do not want to upload their images to the database are considered disadvantages of the system. The importance of collecting more data so that body measurements can be determined more precisely and clearly is emphasized. As a result, this study has developed a more effective and accurate technique than traditional methods and offers potential applications in obesity monitoring and body measurements in the textile industry. In our future studies, more comprehensive studies are planned on natural human bodies within the framework of the anthropometric measurement criteria of the International Society of Kinesiology and Anthropometry (ISAK).

## Data Availability

All data generated or analyzed during this study are included in this published article.
